# A Mendelian randomization study investigating causal links between gut microbiota or metabolites and chronic hepatitis B

**DOI:** 10.3389/fpubh.2024.1398254

**Published:** 2024-07-24

**Authors:** Tongjing Xing, Xuequan Wang, Shanshan He

**Affiliations:** Taizhou Hospital of Zhejiang Province Affiliated to Wenzhou Medical University, Linhai, Zhejiang, China

**Keywords:** chronic hepatitis B, gut microbiota, metabolites, Mendelian randomization analysis, causal links

## Abstract

**Objective:**

This study aimed to explore the potential causal relationship between the gut microbiota and/or its metabolites and the progression of chronic hepatitis B (CHB).

**Method:**

The gut microbiota was used as the exposure factor. The training set exposure data were obtained from the China Nucleotide Sequence Archive (CNSA). Genome-wide association study (GWAS) data from Asia were used as the outcome variables. Outcome data for both the training and validation sets were sourced from the GWAS Catalog database. A dual-sample Mendelian randomization approach was used to analyze the causal relationships, with the inverse variance-weighted method serving as the main analytical strategy. Sensitivity analysis was conducted to assess the robustness of Mendelian randomization analysis results.

**Result:**

In the training set database, analysis using the inverse variance-weighted method revealed a positive correlation between *Fusobacterium varium* and chronic hepatitis B [OR = 1.122, 95% CI (1.016, 1.240), *p* = 0.022]. Conversely, *Veillonella parvula* exhibited a negative correlation with chronic hepatitis B [OR = 0.917, 95% CI (0.852, 0.987), *p* = 0.021]. Sensitivity analysis revealed no evidence of pleiotropy and heterogeneity. No gut microbiota metabolites with a causal effect on chronic hepatitis B were identified. Additionally, no associations between the gut microbiota and the progression of chronic hepatitis B were found in the validation data from the European cohort.

**Conclusion:**

This study suggests that *F. varium* may facilitate the progression of chronic hepatitis B, whereas *V. parvula* may impede it. No causal relationships between gut microbiota metabolites and chronic hepatitis B were established.

## 1 Introduction

Chronic hepatitis B virus (HBV) infection is a serious public health issue that threatens human health. Currently, there are approximately 254 million people worldwide with chronic HBV infection. In China alone, there are an estimated 86 million people with chronic HBV infection and 26 million with active chronic hepatitis B. Between 20 and 40% of chronic hepatitis B patients may die from liver failure, cirrhosis, or hepatocellular carcinoma (1, 2). Chronic persistent HBV infection is primarily related to a weakened HBV-specific immune response. The recurrent occurrence of intrahepatic inflammation and necrosis can lead to the progression of chronic HBV infection to cirrhosis and liver cancer. Antivirus therapy is an effective treatment for chronic hepatitis B. Nucleos(t)ide analogs (NAs) are the most commonly used antivirus drugs, especially the application of highly efficient and low-level drug-resistant NAs, which has greatly reduced the incidence of cirrhosis and liver cancer, significantly improving patient prognoses (3, 4). However, disease progression still occurs in some patients despite long-term NA treatment (5), suggesting that there may be other mechanisms influencing disease progression.

Gut microbiota, the microbial community in the human gut, is closely related to the normal physiological functions of the host. Dysfunction of the gut microbiota plays an important role in the pathogenesis of various diseases, such as gastrointestinal diseases, chronic liver diseases, and tumors (6, 7). Studies have demonstrated significant alterations in the diversity and abundance of gut microbiota in patients with chronic hepatitis B, which may influence their peripheral immunity and disease progression (8, 9). Gut microbiota exhibits specificity at different stages of chronic HBV infection (10). Patients with chronic hepatitis B exhibited a significant reduction in intestinal bifidobacteria and lactobacilli, along with a higher abundance of Enterobacteriaceae and Bacteroidetes (11). Lactic acid bacteria are positively correlated with disease progression, whereas *Clostridium* is negatively correlated (10). Changes in microbial communities are highly correlated with alterations in host metabolism, which in turn may be associated with the progression of chronic hepatitis B. Metabolites derived from the gut microbiota could regulate the host’s immune system (12, 13). Comprehensive analyses of the microbiome and metabolomics have revealed significant changes in the gut microbiota and metabolites in HBV-related chronic liver disease (9, 14). However, most studies have only revealed correlations and have not provided direct causal evidence between these factors.

Mendelian randomization (MR) utilizes genome-wide association studies (GWASs) to obtain single-nucleotide polymorphisms (SNPs) that are strongly correlated with specific outcomes. MR serves as a tool to infer causal relationships between exposure factors and outcomes by using SNPs, which, on the basis of random Mendelian genetic variations, allow for the evaluation of such relationships while minimizing the influence of confounding factors (15). The annotation of SNPs in the gut microbiota, as validated by MR analysis, can reveal relevant genes. Zhang et al. (16) reported that several gut microbiota genera have a causal relationship with the progression of chronic hepatitis B, as determined by MR analysis. However, their study included both European and Asian populations and did not perform cluster analysis. Furthermore, no reports currently exist on MR analysis of gut microbiota metabolites in relation to chronic hepatitis B. In this study, double-sample Mendelian randomization analysis was performed to investigate the causal relationship between the types and abundance of gut microbiota or its metabolites and the progression of chronic hepatitis B, with the effectiveness of the findings validated using multiple methods.

## 2 Materials and methods

### 2.1 Exposure data

We obtained the exposure data for the training set from the China Nucleotide Sequence Archive (CNSA), a convenient and rapid online submission database for biological research projects, samples, experiments, and data. We used the metabolites and gut microbiota data of Asians, as provided in the study with reference (17) from the CNSA database as the training set for exposure data (17).^1^ The exposure data for validation were from the GWAS Catalog database.^2^ As of September 2018, the GWAS Catalog database contains 5,687 GWA entries featuring 71,673 genetic variation phenotype trait associations from 3,567 publications. For our validation set exposure data, we used the European gut microbiota data presented in the publication associated with reference (18).

### 2.2 Outcome data

We sourced the outcome data for the training set from the GWAS Catalog database (see text footnote 2) and the UK BioBank database.^3^ The UK BioBank database was developed by the Medical Research Council Integrative Epidemiology Unit (MRC IEU) OpenGWAS project. The settings of the OpenGWAS database are designed to be scalable and open-source, allowing the importation and publication of complete GWAS summary and summary datasets for use by the scientific community. For the training set outcome data, we used the data with identifiers bbj-a-99 and GCST90018584 pertaining to Asian populations. We also obtained the outcome data for the validation set from the GWAS Catalog database with identifiers GCST90038627, GCST90041715, GCST90041716, and GCST90077701 pertaining to European populations.

### 2.3 Selection of instrumental variables

To ensure the core assumptions of Mendelian randomization, we adhered to the following three criteria during this study. (1) The association hypothesis: genetic variation needs to be strongly associated with the exposure factors. (2) The independence hypothesis: genetic variation is not associated with any possible confounding factors. (3) The exclusivity hypothesis: genetic variation affects the outcomes solely through the exposure factors and not directly. We used a series of conditions to screen the instrumental variables to ensure sufficient instrumental variables. To screen instrumental variables and ensure their adequacy, we selected SNPs (*F* statistics = beta2/se2) with a genome-wide significance level (*p* < 5 × 10^−6^) and *F* statistics ≥10, which indicated a strong correlation with the exposure factors. For the independence test criterion, we tested the selected instrumental variables and set the linkage disequilibrium threshold at *r*^2^ < 0.001 and a genetic distance of 10,000 kb to mitigate the effects of linkage disequilibrium. Finally, to ensure consistency in the allelic directions of the SNP effects on exposure and outcomes, we excluded palindromic SNPs with ambiguous flips, such as those with A/T or G/C alleles.

### 2.4 Mendelian randomization analysis

**Figure 1:**
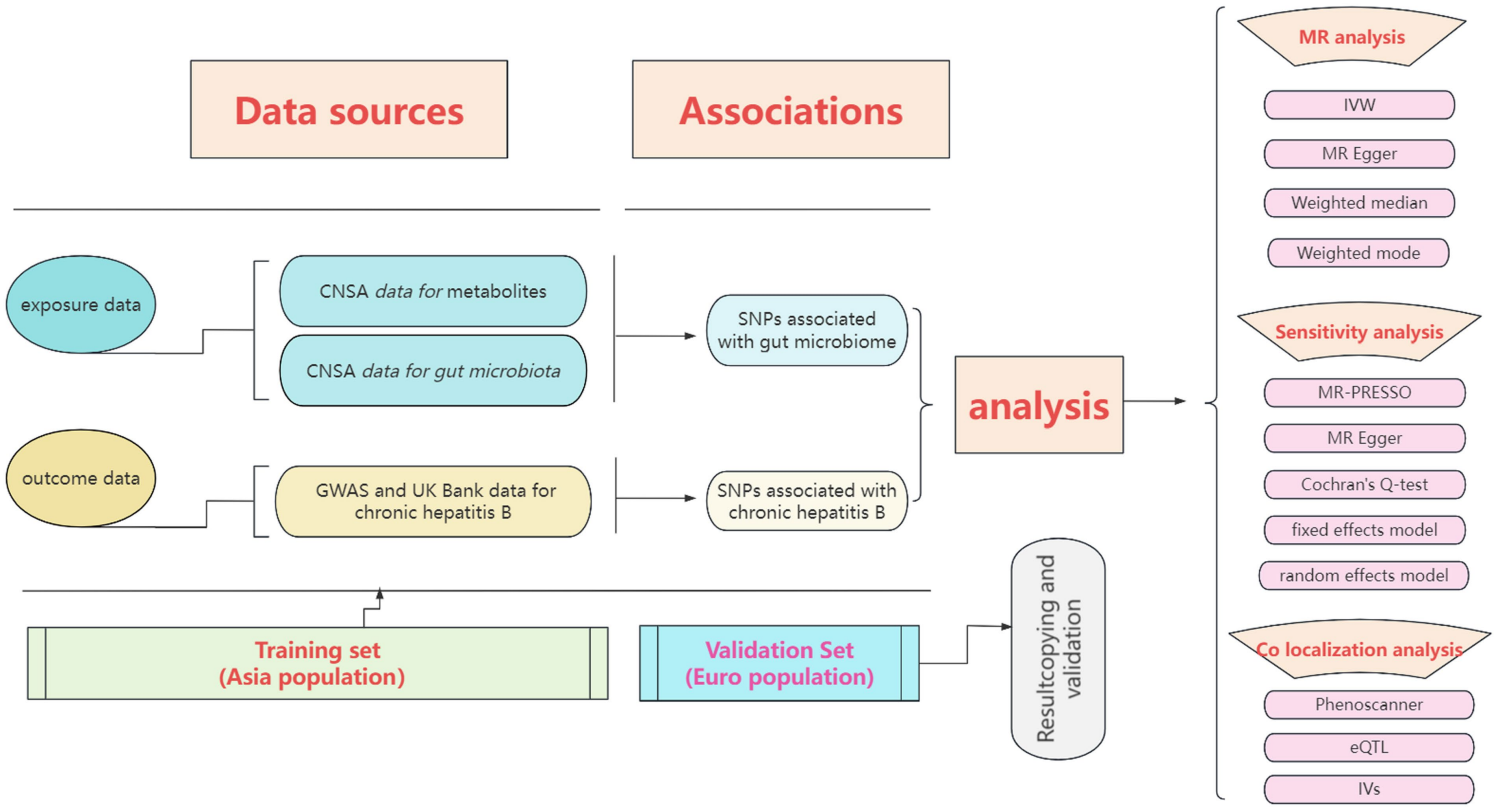
The entire research process of Mendelian randomization analysis.

We conducted a double-sample MR analysis using the inverse variance weighted (IVW), MR Egger, weighted media, simple mode, and weighted mode methods to estimate the effect of exposure factors on outcome data (19). Regarding algorithmic principles, the IVW method integrates the Wald ratio of causal effects for each SNP through meta-analysis to provide the most accurate estimate. Hence, when multiple instrumental variables are used, the IVW method is the primary approach, with the other four methods serving as supplementary analyses. The entire research process is depicted in Figure 1.

### 2.5 Sensitivity analysis

Multiple statistical methods were used for sensitivity analysis. Initially, MR pleiotropy residual sum and outlier tests were conducted to detect horizontal pleiotropy (*p* < 0.05) and remove outlier SNPs (20). Simultaneously, the MR Egger intercept was used to evaluate the level of pleiotropy (21). Subsequently, Cochran’s *Q* test (a heterogeneity test) was used to evaluate the heterogeneity among the instrumental variables. Depending on the degree of heterogeneity, a fixed-effects model was used for IVW calculations when *Q* > 0.05. Conversely, a random-effects model was used when *Q* < 0.05. Further analysis was then conducted using either the fixed-effects model or the random-effects model, as appropriate. Additionally, a one-way analysis was performed to ascertain whether the significant association between exposure factors and outcomes is driven by any individual SNP. This involved sequentially removing different SNPs for MR analysis in each iteration. All the aforementioned analyses, including sensitivity analysis and MR analysis, were performed in the R package (R version 4.2.2) (22).

### 2.6 Colocalization analysis

Colocalization analysis is commonly used to identify whether two phenotypes are influenced by the same causal variant within a certain region, thereby strengthening the evidence of an association between the two phenotypes. After establishing the causal relationship between exposure factors and outcomes, the following methods were used for gene colocalization calculation. (1) Based on PhenoScanner’s exploration of the correlation between expression quantitative trait loci and instrumental variables (IVs) in GTEx data, the IVs related to gene expression were screened with a threshold of *p* < 1e−05. (2) Each IV identified through this screening was treated as lead SNP, and SNPs within the 50 kb range upstream and downstream were examined for colocalization. Gene-level pleiotropy was assessed using a posterior probability threshold of 0.75. This analysis was conducted using the “coloc” package in R.

## 3 Results

### 3.1 SNP selection

After controlling for linkage disequilibrium effects and *F*-statistic values, instrumental variables were extracted from the summary GWAS data of exposure factors. In the positive result, *Veillonella parvula* had the highest number of SNPs, totaling eight, as presented in Table 1. For *Fusobacterium varium*, four SNPs were identified as instrumental variables, which are presented in Table 2.

**Table 1:** SNP of *Veillonella_parvula*.

SNP	effect_allele.exposure	other_allele.exposure	*p* val.exposure	*F*
rs151291166	T	G	3.03E-06	21.96886312
rs117284160	G	T	4.82E-06	21.04684955
rs80119530	A	G	4.30E-06	21.28720553
rs13331430	A	T	6.95E-07	24.83979535
rs144454879	C	T	3.57E-06	21.63556649
rs76339618	C	T	2.93E-06	22.02858508
rs34706036	T	A	4.21E-06	21.3191254
rs9313193	A	G	7.52E-07	24.69731645

**Table 2:** SNP of *Fusobacterium_varium*.

SNP	effect_allele.exposure	other_allele.exposure	*p* val.exposure	*F*_Statistic
rs34292801	C	A	4.12E-06	21.21108025
rs1959910	C	T	2.30E-06	22.33292192
rs4814813	T	G	2.29E-07	26.76913546
rs73088610	T	A	2.81E-06	21.94117388

### 3.2 Mendelian randomization analysis

**Figure 2:**
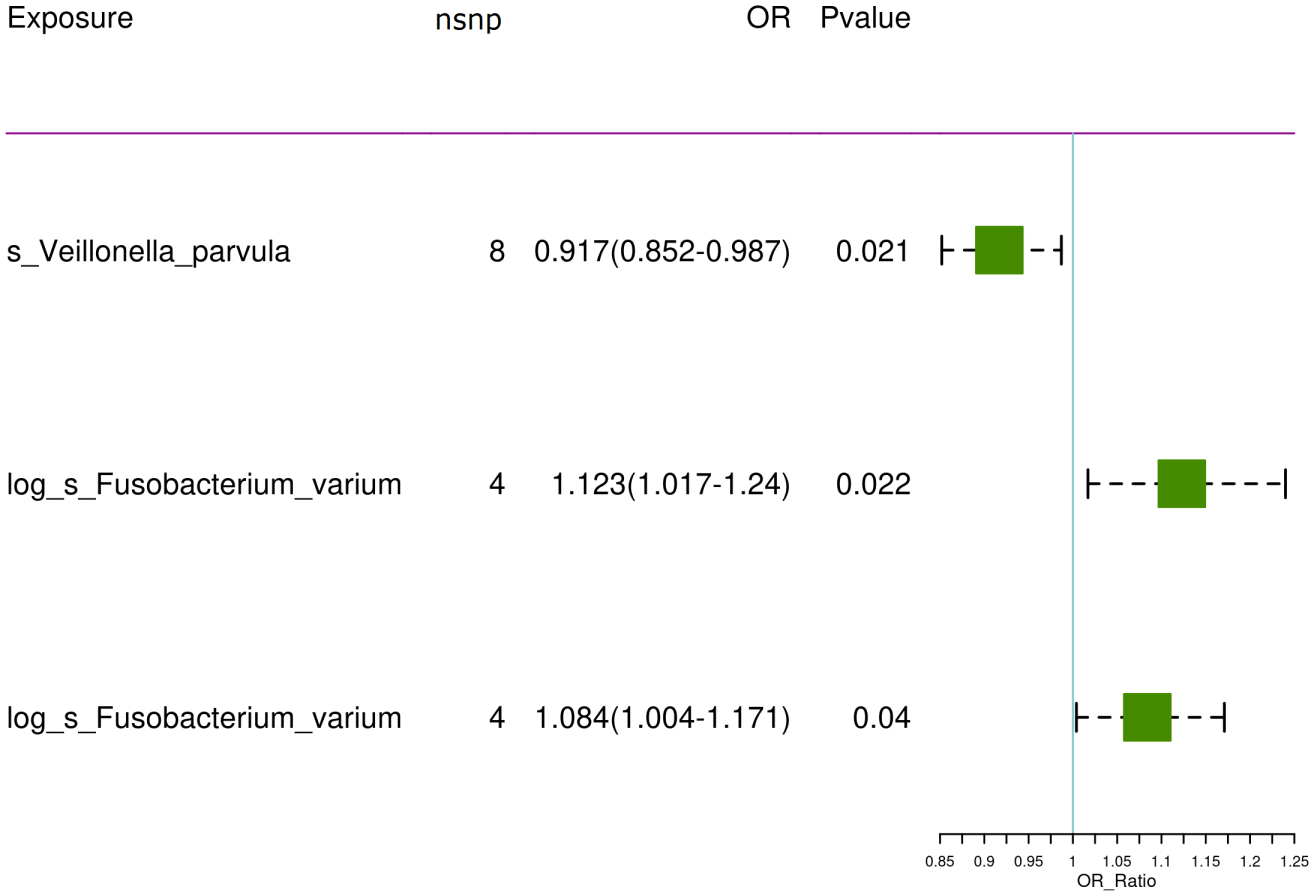
The significant causal relationship between intestinal microbiota and CHB.

**Figure 3:**
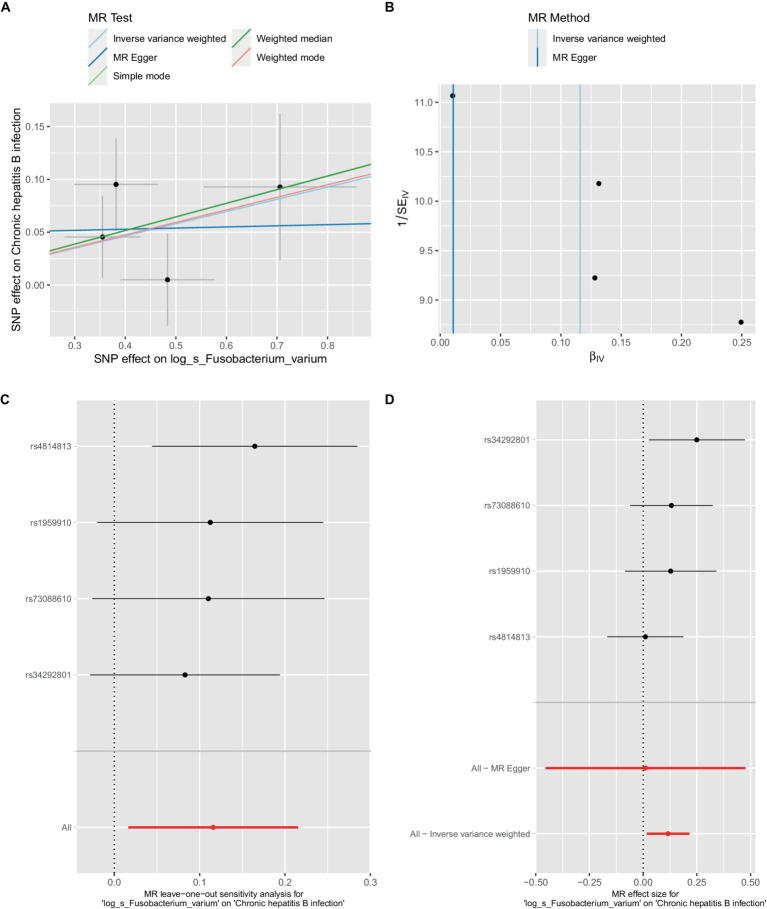
Mendelian randomization analysis between *Fusobacterium*_*varium* and CHB in bbj-a-99. **(A)** IVW model analysis between *F. varium* and CHB. **(B)** Cochran’s Q-test for heterogeneity. **(C)** Analysis of the retention method of single SNP affected on the overall relationship estimation. **(D)** Analysis of effect of individual SNPs on CHB.

**Figure 4:**
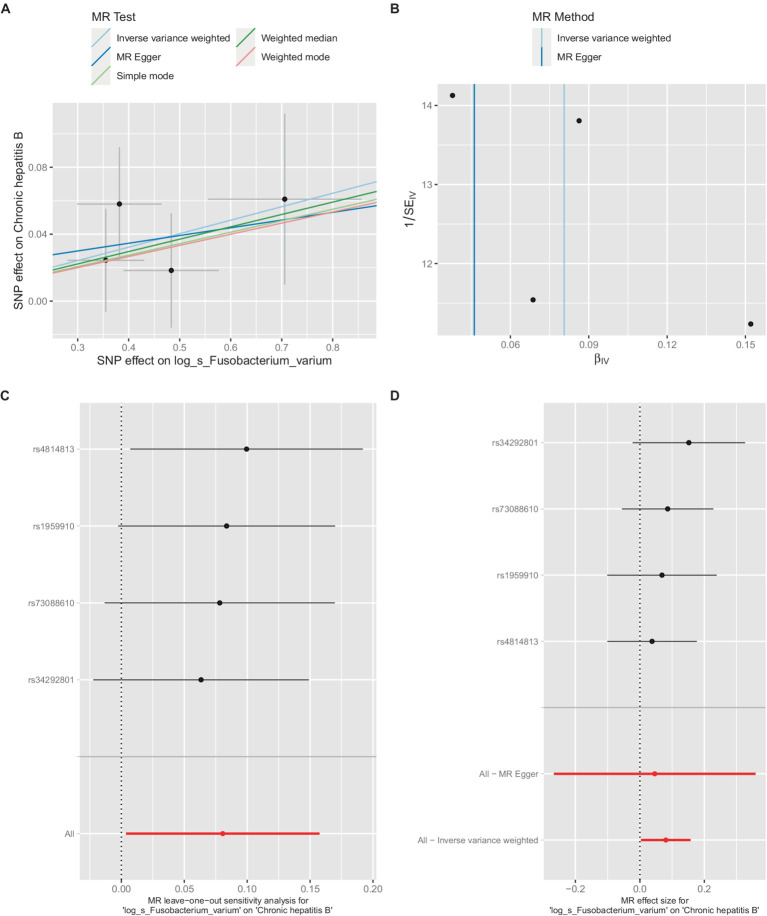
Mendelian randomization analysis between *Fusobacterium_varium* and CHB in GCST90018584. **(A)** IVW model analysis between *Fusobacterium*_*varium* and CHB. **(B)** Cochran’s Q-test for heterogeneity. **(C)** Analysis of the retention method of single SNP affected on the overall relationship estimation. **(D)** Analysis of effect of individual SNPs on CHB.

For the training set exposure data and training set outcome data, one-on-one MR analyses were conducted. Within the training set, IVW analysis indicated causal relationships between two types of gut microbiota and chronic hepatitis B at a significance threshold of 5 × 10^−6^ as presented in Table 3 (Figure 2). *F.* var*ium* exhibited a promotive effect in both datasets analyzed, whereas *V. parvula* demonstrated an inhibitory effect. Results from the MR Egger intercept and MR pleiotropy residual sum and outlier pleiotropy tests indicated no pleiotropy (Supplementary Table S1). The results of the MR analysis are presented in Figures 2–4.

**Table 3:** Intestinal microbiota with significant causal effects on CHB.

	Method	*p* val	OR	OR_LCI95	OR_UCI95
s_Fusobacterium_varium_bbj-a-99	Inverse variance weighted	0.0224	1.1229	1.0166	1.2404
	MR Egger	0.9679	1.0108	0.6347	1.6098
	Simple mode	0.2554	1.138	0.9499	1.3634
	Weighted median	0.0411	1.1375	1.0052	1.2872
	Weighted mode	0.2632	1.1259	0.9507	1.3335
s_Fusobacterium_varium_GCST90018584	Inverse variance weighted	0.04	1.0839	1.0037	1.1706
	MR Egger	0.7996	1.0473	0.7659	1.4321
	Simple mode	0.3373	1.071	0.9518	1.2052
	Weighted median	0.108	1.0768	0.9839	1.1784
	Weighted mode	0.3518	1.0688	0.9492	1.2035
s_Veillonella_parvula_GCST90018584	Inverse variance weighted	0.0214	0.9171	0.8519	0.9873
	MR Egger	0.391	0.8902	0.7022	1.1285
	Simple mode	0.0931	0.8671	0.7576	0.9924
	Weighted median	0.0335	0.8943	0.8068	0.9913
	Weighted mode	0.0972	0.8914	0.7982	0.9956

In the bbi-a-99 dataset, the IVW model analysis revealed a significant causal relationship between *F.* var*ium* and chronic hepatitis B [OR = 1.1229, 95% CI (1.0166, 1.2404), *p* = 0.0224] (Figure 3A). Cochran’s *Q*-test results indicated no heterogeneity (Figure 3B). The analysis results of the retention method suggested that the overall relationship estimation is not significantly affected by any single SNP (Figure 3C). Analysis of individual SNPs revealed that some SNPs may confer a protective effect against chronic hepatitis B (Figure 3D).

Similarly, in the GCST90018584 dataset, IVW model results confirmed a significant causal relationship between *F. varium* and chronic hepatitis B [OR = 1.0839, 95% CI (1.0037, 1.1706), *p* = 0.04] (Figure 4A), with Cochran’s *Q*-test results indicating no heterogeneity (Figure 4B). The results of the retention method analysis indicated that the overall relationship estimate was not significantly affected by any single SNP (Figure 4C), whereas single-SNP analysis suggested a certain protective trend against chronic hepatitis B (Figure 4D).

**Figure 5:**
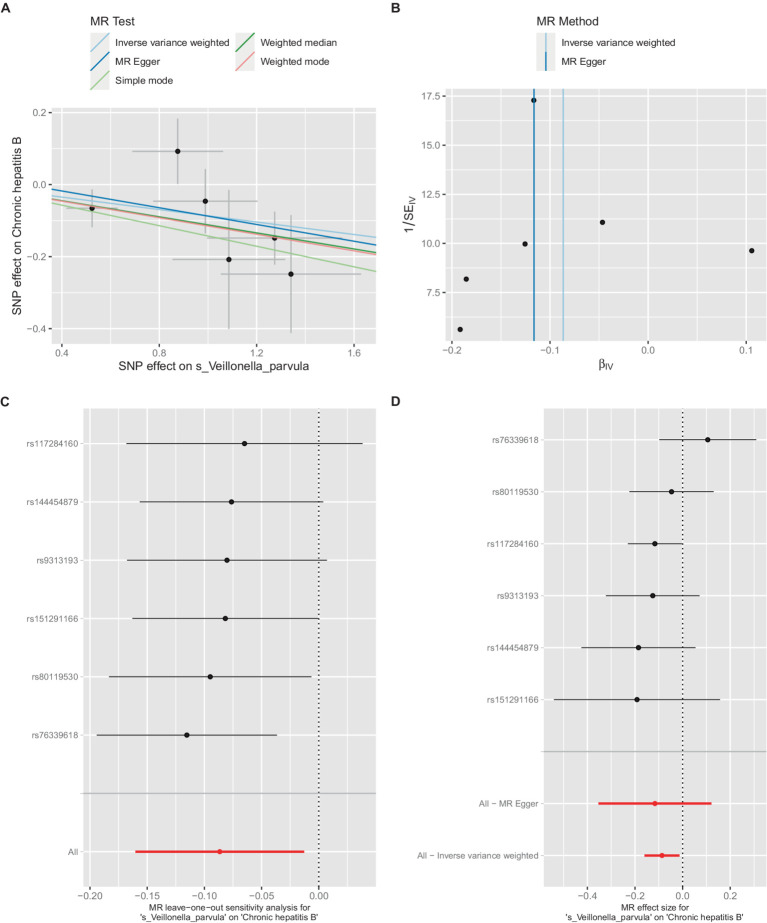
Mendelian randomization analysis between *Veillonella_parvula* and CHB in GCST90018584. **(A)** IVW model analysis between *Veillonella*_*parvul* and CHB. **(B)** Cochran’s Q-test for heterogeneity. **(C)** Analysis of the retention method of single SNP affected on the overall relationship estimation. **(D)** Analysis of effect of individual SNPs on CHB.

Additionally, within the GCST90018584 dataset, IVW model results indicated a significant causal relationship between *V. parvula* and chronic hepatitis B [OR = 0.9171, 95% CI (0.8519, 0.9873), *p* = 0.0214] (Figure 5A), with Cochran’s *Q*-test results indicating no heterogeneity (Figure 5B). The results of the retention method analysis indicated that the overall relationship estimation is not significantly affected by any single SNP (Figure 5C). Single-SNP analysis indicated a certain protective effect against chronic hepatitis B (Figure 5D).

### 3.3 Colocalization analysis

Colocalization analysis was performed on two types of bacteria, one of which was analyzed in two datasets (bbj-a-99 and GCST90018584). The correlation between *Fusobacterium* expression level and four SNPs was explored using PhenoScanner within GTEx data. No significant correlation was found between them in the bbj-a-99 datasets. Colocalization analysis within the 50-kb range upstream and downstream of these SNP sites indicated that the causal relationships do not exhibit gene-level pleiotropy, with a negative threshold of 0.75 (Table 4). Similarly, in the GCST90018584 dataset, no significant correlation was observed between *Fusobacterium* expression levels and the four SNPs (Table 5). The analysis of *Veillonella* expression level and six SNPs using PhenoScanner within GTEx data also indicated no significant correlation in the GCST90018584 dataset. Colocalization analysis of these SNP sites indicated a lack of gene-level pleiotropy with a negative threshold of 0.75 (Table 6).

**Table 4:** Co localization results of *Fusobacterium_varium* in bbj-a-99.

SNP	PP.H0.abf	PP.H1.abf	PP.H2.abf	PP.H3.abf	PP.H4.abf
rs1959910	0.04701251	0.8996013	0.001194543	0.022828634	0.02936303
rs34292801	0.44627163	0.4694337	0.004631531	0.004797047	0.07486609
rs4814813	0.01318329	0.9613237	0.000117324	0.008538425	0.01683722
rs73088610	0.45742471	0.50087	0.005321152	0.005795956	0.03058819

**Table 5:** Co localization results of *Fusobacterium_varium* in GCST90018584.

SNP	PP.H0.abf	PP.H1.abf	PP.H2.abf	PP.H3.abf	PP.H4.abf
rs1959910	0.04808632	0.9048905	0.001337023	0.025139612	0.02054654
rs34292801	0.46447663	0.4899215	0.006157673	0.006462019	0.03298217
rs4814813	0.01312701	0.956298	0.000171904	0.012505231	0.01789786
rs73088610	0.45102883	0.4911956	0.015389112	0.01673395	0.02565252

**Table 6:** Co localization results of *Veillonella_parvula* in GCST90018584.

SNP	PP.H0.abf	PP.H1.abf	PP.H2.abf	PP.H3.abf	PP.H4.abf
rs117284160	0.9575255	0.02272465	0.01655607	0.000390116	0.002803713
rs144454879	0.9565484	0.02951278	0.011473823	0.000351894	0.002113103
rs151291166	0.9497989	0.03768836	0.009313298	0.000366722	0.002832719
rs76339618	0.8681296	0.11954474	0.006003488	0.000821202	0.005500947
rs80119530	0.7050518	0.03481255	0.236885348	0.011684854	0.011565421
rs9313193	0.1679168	0.7587915	0.002791675	0.012557225	0.057942757

### 3.4 Validation of positive phenotype in the European population dataset

Based on the positive phenotype of the training set, data related to relevant phenotypes in the European population were obtained from the literature (PMID: 35115689) to serve as exposure data for the validation set of gut microbiota. Datasets GCST90038627, GCST90041715, GCST90041716, and GCST90077701 were used as outcome data for the validation set. No positive results were observed in the European population dataset (Supplementary Table S2).

## 4 Discussion

The liver, as the principal immune organ, processes a large amount of gut-derived components and toxins to maintain immune homeostasis. Disruptions in the equilibrium of the gut microbiota are implicated in a spectrum of liver diseases (23). Gut microbiota imbalance is characterized by an increased proportions of *Bacillus* and *Proteus*, and a decreased proportions of *Clostridium* associated with the onset of bacterial translocation. These microorganisms infiltrate the bloodstream via the portal vein and then enter the liver tissue, where they activate Kupffer cells and hepatic stellate cells through Toll-like receptor 4, thereby promoting disease progression (24). Investigating the causal relationship between the gut microbiota, its metabolites, and the development of chronic hepatitis B may provide further insight into the pathogenesis of the disease and personalized approaches to microbiome modulation (25). In this study, we evaluated the causal relationship between the gut microbiota, its metabolites, and the advancement of chronic hepatitis B through MR analysis using publicly available GWAS summary data. Our findings indicate that *F.* var*ium* exerts a promoting effect, whereas *V. parvula* exhibits an inhibitory effect. These outcomes diverge from those reported by Zhang et al. (16). Possible reasons may be related to factors such as the selected population and the diversity of gut microbiota. No metabolites were identified as having a causal relationship with the progression of chronic hepatitis B.

*Fusobacterium* is one of the symbiotic bacteria in humans and animals primarily mainly colonizing the oral and colonic mucosa. Some species, which are opportunistic pathogens, can cause bacteremia and various rapidly progressing infections. These microorganisms are more toxic than most normal anaerobic bacterial communities, as they produce endotoxins and hemolysin. *Fusobacterium* can trigger pro-inflammatory responses in the host and possesses virulence characteristics that promote adhesion to host epithelial cells and invasion of epithelial cells (26). Research has indicated that *Fusobacterium* may be associated with intestinal diseases. The concentration of *Clostridium* in colon cancer tissue is significantly higher than that in normal tissue, which may contribute to tumor development. *Fusobacterium* is also associated with inflammatory bowel disease and intestinal infections (27, 28). In the oral cavity, *Fusobacterium* is associated with oral diseases such as dental caries and periodontitis. However, *Fusobacterium* may also play a role in maintaining the homeostasis and metabolic function of the gut microbiota and can produce beneficial metabolites for the human body, such as pyruvate and butyric acid. The study by Liu et al. reported alterations in the microbial composition and function in patients with cerebral autosomal dominant arteriopathy with subcortical infarcts and leukoencephalopathy by multi-omics studies (29). The abundance of mutated *F.* var*ium* positively correlated with IL-1 β and IL-6 levels in patients with cerebral autosomal dominant arteriopathy with subcortical infarcts and leukoencephalopathy. This study indicates that *F. varium* may have a deleterious effect on the progression of chronic hepatitis B disease, suggesting that *F. varium* may promote the progression of chronic hepatitis B disease, potentially due to its role in elevating levels of inflammatory factors.

*Veillonella* belongs to the genus Gram-negative anaerobic *Micrococcus*, with more than 10 known within the genus. Recent research has revealed that *Veillonella* significantly affects the human microbiome, infection processes, and immune system development (30). Qin et al. found an increase in *Veillonella* abundance among the gut microbiota of patients with liver cirrhosis (31). Wei et al. reported that an increase in *Veillonella* abundance is strongly associated with autoimmune hepatitis and is positively correlated with serum AST and liver inflammation (32). *V. parvula*, isolated from patients with appendiceal abscess, has been found in multiple ecological niches within the human body, including the mouth, lungs, gastrointestinal tract, and vagina. Rojas et al. (33) found that *V. parvula* colonizes the intestine by using alterations in carbon metabolism and ATP production pathways during inflammation. The presence of *V. parvula* is associated with disease remission and stability. These specific bacterial genera may be biomarkers in response to immune checkpoint inhibitor therapy (34). Jin et al. (35) demonstrated that an increase in intestinal *V. parvula* induced liver cell damage, hepatic stellate cell activation, and subsequent progression of cirrhosis by interacting with the TLR4/NLRP3 signaling pathway in a mouse model. This study indicates that *V. parvula* has an inhibitory effect on chronic hepatitis B, suggesting that *V. parvula* may inhibit the progression of chronic hepatitis B.

The metabolites produced by the gut microbiota serve as signaling molecules and substrates, affecting both pathological and physiological processes. Many beneficial microorganisms, such as rumen bacteria, fecal bacteria, *Clostridium*, and *Prevotella*, are known to play important roles in enhancing short-chain fatty acid activity and increasing butyric acid abundance. The study of Li et al. identified 134 named metabolites (57 upregulated and 77 downregulated metabolites) in patients with chronic hepatitis B. This study predicted a total of 101 different metabolic functions with six metabolic pathways having the highest enrichments (14). The study of Zeng et al. (36) reported that certain fecal metabolites such as essential amino acids and several dipeptides may be associated with HBeAg seroconversion of chronic hepatitis B patients. These findings provided evidence for changes in gut microbiota–related metabolites in patients with chronic HBV infection. However, the causal relationship between gut metabolites and chronic hepatitis B has not yet been established. Our results indicated that there is no causal relationship between serum metabolites related to gut microbiota and the progression of chronic hepatitis B. These results indicate that although gut microbiota–related metabolites exhibit significant differences between patients with chronic hepatitis B and healthy individuals, their effect on the progression of chronic hepatitis B may be minimal or inconsequential. The presence of a specific bacterial genus or metabolite alone may not be sufficient to trigger disease progression. This could also relate to the typically prolonged natural course of chronic liver disease in humans, which often spans years or decades. Nevertheless, the potential synergistic effect of multiple metabolites or lower abundance of certain fecal metabolites on the progression of chronic hepatitis B cannot be completely discounted, warranting further in-depth investigation.

This study has several limitations. First, our MR analysis restricted by GWAS data was limited to the genus level and not the species level. Second, training group and validation group populations from different races may be affected by genetic variation. Additionally, longitudinal research was not performed in this study. Further longitudinal studies are required to validate our results in the population with chronic hepatitis B in the future.

In summary, this study indicates that *F. varium* may have a promoting effect, whereas *V. parvula* (*Vibrio* genus) appears to have an inhibitory effect. However, no gut microbiota metabolites have been definitively found to have a causal relationship with the progression of chronic hepatitis B. This provides new evidence for elucidating the potential relationships between the gut microbiota, its metabolites, and chronic hepatitis B.

## Data availability statement

The datasets presented in this study can be found in online repositories. The names of the repository/repositories and accession number(s) can be found in the article/Supplementary material.

## Author contributions

TX: Writing – original draft. XW: Writing – review & editing. SH: Writing – review & editing.

## Footnotes

^1^ https://ftp.cngb.org/pub/CNSA/data2/CNP0000794/

^2^ https://www.ebi.ac.uk/gwas/

^3^ https://gwas.mrcieu.ac.uk/
