# Supplementary material for: A Mendelian randomization study investigating causal links between gut microbiota or metabolites and chronic hepatitis B
Source: Front Public Health. 2024 Jul 24;12:1398254. doi: 10.3389/fpubh.2024.1398254 (PMC11303287; doi:10.3389/fpubh.2024.1398254)
Supplement: Supplementary file 1 [file Table_1.DOCX]

**Table S1**  Multipotency testing of three microbial communities

|  | MR_Presso_Pvalue | MR_Egger_interscept | MR_Egger_Pvalue |
| --- | --- | --- | --- |
| Fusobacterium_variuml-bbj-a-99 | 0.497 | 0.0485 | 0.693 |
| Fusobacterium_variuml-GCST90018584 | 0.81 | 0.0161 | 0.8447 |
| Veillonella_parvul-GCST | 0.51 | 0.0289 | 0.8066 |
